# Patient perspectives on quality of care for depression and anxiety in primary health care teams: A qualitative study

**DOI:** 10.1111/hex.13242

**Published:** 2021-05-05

**Authors:** Rachelle Ashcroft, Matthew Menear, Andrea Greenblatt, Jose Silveira, Simone Dahrouge, Nadiya Sunderji, Monica Emode, Jocelyn Booton, Marvelous Muchenje, Rachel Cooper, Asante Haughton, Kwame McKenzie

**Affiliations:** ^1^ Factor‐Inwentash Faculty of Social Work University of Toronto Toronto ON Canada; ^2^ Faculty of Medicine Department of Family Medicine and Emergency Medicine Université Laval Quebec Quebec Canada; ^3^ Faculty of Medicine Department of Psychiatry University of Toronto Toronto ON Canada; ^4^ Faculty of Medicine Department of Family Medicine University of Ottawa Ottawa ON Canada; ^5^ Faculty of Medicine Department of Psychiatry Institute for Health Policy, Management and Evaluation Dalla Lana School of Public Health University of Toronto Toronto ON Canada; ^6^ School of Population and Public Health University of British Columbia Vancouver Canada; ^7^ Center for Bioethics Harvard Medical School Boston MA USA; ^8^ Stella’s Place Toronto ON Canada; ^9^ Faculty of Medicine, Department of Psychiatry, University of Toronto I Wellesley Institute Toronto ON Canada

**Keywords:** interprofessional teams, mental health care, patient engagement, primary care, quality of care

## Abstract

**Background:**

Widespread policy reforms in Canada, the United States and elsewhere over the last two decades strengthened team models of primary care by bringing together family physicians and nurse practitioners with a range of mental health and other interdisciplinary providers. Understanding how patients with depression and anxiety experience newer team‐based models of care delivery is essential to explore whether the intended impact of these reforms is achieved, identify gaps that remain and provide direction on strengthening the quality of mental health care.

**Objective:**

The main study objective was to understand patients’ perspectives on the quality of care that they received for anxiety and depression in primary care teams.

**Methods:**

This was a qualitative study, informed by constructivist grounded theory. We conducted focus groups and individual interviews with primary care patients about their experiences with mental health care. Focus groups and individual interviews were recorded and transcribed verbatim. Grounded theory guided an inductive analysis of the data.

**Results:**

Forty patients participated in the study: 31 participated in one of four focus groups, and nine completed an individual interview. Participants in our study described their experiences with mental health care across four themes: accessibility, technical care, trusting relationships and meeting diverse needs.

**Conclusion:**

Greater attention by policymakers is needed to strengthen integrated collaborative practices in primary care so that patients have similar access to mental health services across different primary care practices, and smoother continuity of care across sectors. The research team is comprised of individuals with lived experience of mental health who have participated in all aspects of the research process.

## 1 INTRODUCTION

In most countries, primary care is the first point of contact in the health‐care system for many individuals with mental health problems.^1^ Increasingly, interprofessional primary care teams are optimally positioned to address specific mental health needs of patients along with other physical and/ behavioural needs.^2^, ^3^ Given the high prevalence of common mental disorders (CMDs)—such as anxiety and depression—in primary care^1^ and the challenges of clinical management, there is substantial benefit from the collaboration between health and mental health professionals who can work together as a team.^4^, ^5^, ^6^, ^7^, ^8^, ^9^

The Patient Medical Home (PMH) is a team‐based model of primary care that has continued to accelerate over the last two decades in Canada, the United States and elsewhere.^10^, ^11^, ^12^, ^13^ In PMHs, family physicians work in tandem with interprofessional teams to provide continuous and coordinated person‐centred care.^10^, ^11^, ^12^, ^13^ The PMH is an optimal model for the integration of effective high‐quality mental health care.^11^ In Ontario, Canada, the implementation of a PMH model of primary care had consequences for people living with mental health problems.^12^ This includes reforms beginning in the early 2000s involving a shift away from fee‐for‐service–based physician remuneration to a capitation‐based system, and the expansion of collaborative team–based care mainly through the creation of Family Health Teams (FHTs).^12^, ^13^ FHTs bring family physicians together with nurses, nurse practitioners, social workers, mental health counsellors, pharmacists, dieticians, consulting psychiatrists and other health‐care professionals.^12^, ^13^ Currently, there are 186 FHTs serving approximately three million Ontarians (22% of the provincial population).^12^, ^13^ FHTs were established with intention to improve access to comprehensive person‐centred care, and improve continuity of care with other parts of the health‐care system.^10^, ^11^, ^12^, ^13^, ^14^, ^15^

FHTs had also anticipated benefits for patients requiring mental health services for CMDs.^2^ The integration of teams in primary care is advantageous for patients who can then access a range of physical and mental health services in one location,^2^, ^16^ with shorter wait times for mental health services than traditional care settings.^2^, ^17^, ^18^, ^19^ Embedding mental health services in the same organization where patients see their family physician—someone with whom they have a long‐standing trusting relationship—may help reduce stigmatization.^2^ By reducing stigmatization, patients may be more willing to seek out mental health services when needed, especially when the mental health provider is someone who works in tandem with their family physician.^2^, ^20^, ^21^ FHTs host a number of mental health practitioners, including social workers (92% of FHTs), psychologists (25%) and other mental health workers (13%).^22^ Combining these providers and others, with family physicians, improves prevention and enhances identification, early intervention and treatment while improving patient experiences.^2^, ^22^, ^23^, ^24^, ^25^ Despite the increasing emphasis of primary care teams, we know little about the impact of this team structure on patient experiences with mental health care.^2^, ^26^

There is overwhelming evidence that knowledge about approaches to delivering effective mental health services is not consistently translated into action^26^ with many people continuing to struggle with unmet mental health needs.^14^, ^15^, ^16^, ^17^, ^18^, ^19^, ^20^, ^21^, ^22^, ^23^, ^24^, ^25^, ^26^, ^27^, ^28^, ^29^, ^30^, ^31^, ^32^ It is essential to examine the impact of reforms intended to improve mental health‐care delivery from the perspective of the service recipient to inform measures that can drive services that are most meaningful to patients.^27^, ^33^ Understanding how patients experience new models of care delivery is essential to evaluate whether the intended impact of these reforms is achieved, identify gaps that remain and determine whether the new approach resulted in any unintended consequences.^26^, ^33^ Our main study objective was to understand patients’ perspectives on the quality of care that they received for CMDs from Ontario's FHTs.

## 2 METHODS

### 2.1 Study design

We used constructivist grounded theory to guide sampling, data collection and data analyses.^34^, ^35^ Constructivist grounded theory views knowledge as socially constructed, and emphasizes research that recognizes multiple viewpoints and interpretive understandings.^34^, ^35^, ^36^, ^37^, ^38^ It also acknowledges the subjectivity of researchers whereby their assumptions are considered to be valuable for shaping data collection and analysis.^34^, ^36^ Team members involved in this study had different clinical or disciplinary backgrounds spanning: social work, psychiatry, mental health research, epidemiology and primary care health services delivery research. Four members of the team had experience as advisors to provincial policy and decision‐makers. Integral to our team were two individuals with lived experience of CMDs who contributed to the research process, interview questions and interpretation of results. Sensitizing concepts acted as a starting point help to inform the research process, and provide a way of understanding and organizing participant experience.^37^, ^38^, ^39^ The sensitizing concepts were derived through a review of the literature, from lived experience of team members and from our previous research.^27^, ^40^, ^41^, ^42^, ^43^ The data that support the findings of this study are not shared in a public repository. This study received Research Ethics Board Approval from the University of Toronto (REB#35131).

### 2.2 Sampling and recruitment

We sought to engage a diversity of perspectives of people receiving care from FHTs within three different geographical regions of Ontario: Toronto Central, Central East and South East. There were 38 FHTs operating within these three regions, and these varied in terms of team size, provider composition and geographical characteristics. We selected these regions for three reasons: i) regional variation in terms of rural and urban; ii) varying diversity of populations in these regions; and, iii) existing relationships in our team with many FHTs in these regions from a previous study.^38^ Potential participants self‐identified as residing in one of these three regions, and self‐identified as someone who is a patient of a FHT. With the assistance of FHTs and the Canadian Mental Health Association, we recruited participants through flyers posted in waiting rooms and through word of mouth. Potential participants contacted the research coordinator to indicate interest in participating in the study. Eligible participants i) self‐identified as having a diagnosis of CMD; ii) had received service for depression and/or anxiety at a FHT in the designated region; and iii) were adults 18 years and older. Recruitment began in August 2018 and ended in March 2019. After completing the fourth focus group and analysis of the data, we ceased recruitment because theoretical saturation had been reached.^34^ Theoretical saturation occurs when ‘fresh data no longer sparks new theoretical insights, nor reveals new properties of these core theoretical categories’.^34^ ^, p.213^

### 2.3 Data collection

We developed a semi‐structured interview guide using sensitizing concepts. We then collected data using in‐person focus groups. We chose to use focus groups for data collection because of the deep understanding of patients’ perspectives that can emerge from the dynamic nature of focus groups.^44^, ^45^ Focus groups also help generate diverse views and experiences.^46^ We offered individual interviews to those unable to attend a focus group. Two team members co‐facilitated the focus groups and conducted the individual interviews between August 2018 and March 2019. We audio‐recorded focus group and individual interviews, and transcribed verbatim immediately following the interview. We randomly assigned a code to each participant for anonymity.

### 2.4 Data analysis

Analysis began immediately following the transcription of each focus group or interview. Data collection and data analysis occurred simultaneously, resulting in an iterative analysis approach.^45^ The iterative analysis approach helped to inform the on‐going refinement of our interview guide. For example, there were little data in the early interviews and first focus group related to diversity and quality of care, so we added a question and probes about diversity for later interviews and the three latter focus groups. Grounded theory informed initial line‐by‐line coding, followed by focused and axial coding.^34^, ^45^ Two team members parallel‐coded transcripts until they reached consensus in the coding process, after which one member was the primary, and the other was the secondary coder. A data analysis subcommittee met regularly to help interpret the data, discuss emerging findings, inform new coding and update the interview guide based on the emerging findings. A final interpretation of findings included all research team members. We conducted the data analysis inductively. We identified exemplar quotes as analysis proceeded. It was through prolonged engagement, reflexivity and peer debriefing that we established rigour and trustworthiness.^45^, ^47^, ^48^ We used NVivo 11 to help organize the data analysis process.

## 3 RESULTS

### 3.1 Sample

Forty patients participated in the study: 31 participated in one of four focus groups, and nine completed an individual interview (Table 1). The participants in our sample varied by age, ethnicity, gender and geographical location (Tables 2, 3).

**TABLE 1:** Number of participants in four focus groups and individual interviews conducted in three geographical regions

Geographical region	No. of participants per focus group	No. of participant interviews	Total participants
South East	8	7	15 (38%)
Central East	10	1	11 (28%)
Toronto Central	4 + 9	1	14 (35%)
Total	n = 31	n = 9	N = 40

**TABLE 2:** Demographics of participants in each of the four focus groups

Focus group number	Focus group date	Geographical region	Participant age	Participant gender	Participant ethnicity
1	August 2018	South East	33	Female	Caucasian
			52	Female	Caucasian
			60	Female	Caucasian
			41	Male	Multiracial
			56	Male	Métis
			57	Female	Caucasian
			63	Female	Not specified
			45	Female	Caucasian
2	November 2018	Central East	61	Male	Caucasian
			54	Female	Asian
			61	Female	South Asian
			42	Female	Caucasian
			41	Female	South Asian
			60	Female	Caucasian
			53	Female	South Asian
			51	Male	Caucasian
			61	Female	South Asian
			55	Female	Asian
3	January 2019	Toronto Central	68	Female	Not specified
			30	Female	Caucasian
			32	Male	Caucasian
			43	Female	Indigenous
4	March 2019	Toronto Central	57	Female	Asian
			56	Female	Caucasian
			54	Female	Hispanic
			42	Male	Black/Afro‐Caribbean
			59	Male	Caucasian
			46	Female	Caucasian
			43	Female	Black/Afro‐Caribbean
			35	Female	Not specified
			55	Female	Indigenous

**TABLE 3:** All participant demographics: age, ethnicity and gender

Demographics	Number of participants (%)
Age (y)	
18‐29	2 (5%)
30‐39	6 (15%)
40‐49	11 (28%)
50‐59	14 (35%)
60‐69	7 (18%)
Ethnicity	
Caucasian	21 (53%)
South Asian	5 (13%)
Asian	4 (10%)
Indigenous/Métis	3 (8%)
Black/Afro‐Caribbean	2 (5%)
Multiracial or not specified	5 (13%)
Gender^a^	
Male	10
Female	30

^a^ Non‐binary gender options were included on demographic form.

With respect to the care experiences of patients with CMDs and areas of improvement, the following four themes emerged: “accessibility, technical care, trusting relationships and meeting diverse needs”.

### 3.2 Accessibility

There was consensus across all focus groups and individual interviews about the importance of accessible care. Access emerged early in all focus groups and individual interviews, with robust discussion in focus groups about the benefits of embedding mental health professionals in primary care. With mental health professionals integrated into primary care, participants were easily able to access a range multiple mental health services: ‘I’ve seen the therapist…a psychiatrist, psychologist, and then obviously my family doctor’ (I1). All focus groups agreed that the team‐based approach enhanced access: ‘The whole thing about being a health team for me, too, was just the familiarity, the easy access’ (FG1, P6). Facilitating access was particularly important for patients struggling with mental health: ‘Everything is centralized, so as someone who deals with mental health, I don't have to be running around and, especially [when]…mentally exhausted, physically exhausted’ (I4). All focus groups emphasized how important it was that primary care teams enabled access to mental health services at no cost to patients: ‘Cost is a factor for sure…this is free…. That's a huge plus! I don't have benefits’ (FG1, P6). Across all focus groups, participants spoke about how mental health services in primary care made it easier to seek supports because it reduced concerns about stigma. ‘I think some of this stigma thing to accessing…mental health services… nobody knows why you're coming here…I might be seeing my diabetic [nurse]’ (FG1, P6). There was extensive discussion about stigma in the first focus group conducted in a rural community: ‘I think it's…reducing that stigma…because most people don't want to talk about or disclose that they're struggling…that that kind of [stigmatizing] mentality is still embedded deep in our community’ (FG1, P1).

Several barriers to access emerged in each of the focus groups. During the focus groups, participants became acutely aware that there was a different level of awareness about the range of services available in FHTs. Some participants were informed, whereas others were not: ‘They don't tell you these are all the professionals that work here…that you have access to’ (FG3, P3). The dynamic exchange in focus groups brought about an awareness of the variations in the types and amount of services that existed across FHTs. All focus groups and some individual interviews perceived the length of appointments with physicians as inadequate: ‘In the case of new medication… we can look together to see what might be a better fit and you can't necessarily do that if you are allotted 15 minutes’ (I4). All focus groups viewed the caps on the number of therapy sessions as problematic. ‘It was like, “I can only see you for like six times?”…So why do I want to even start this?’ (FG3, P2).

### 3.3 Technical care

#### 3.3.1 Identification and diagnosis

The process of identification and diagnosis emerged in all focus groups and some individual interviews. Many reported that they had initiated the first conversation about mental health with their family physician. ‘I brought it up because I was suffering with suicidal thoughts and stuff like that, so I brought it up’ (I1). One focus group engaged in an in‐depth discussion about various reasons why patients might be hesitant to initiate such a conversation: ‘When I go at 9:15 in the morning they're already behind…They're not going to want to delve into big psychological issues’ (FG4, P7). All focus groups expressed a strong desire to have mental health screening and assessments routinely integrated into their primary care. A participant in one focus group stated, ‘They always do a physical and they take your blood and they check off that it happens but they never…sit down and say “how's your mental health really going?”’ (FG3, P1). Another replied, ‘There's a schedule for when you're supposed to get a PAP test and they'll call you about that, and get you to come in for that but there's not a schedule for mental health check‐ups in the same way’ (FG3, P3). There was agreement that the process of diagnosis and treatment planning needed to occur at a pace that facilitated shared decision making, ‘It was good in the sense that they were trying to find a solution but…sometimes you have to hear it all before you can kind of make a decision’ (I3). One focus group in particular spoke extensively about the need for primary care providers to talk about mental health in a way that is more meaningful to patients: ‘They go more to what they have tangible and factual information on, rather than diving into the mental health area’ (FG4, P9).

#### 3.3.2 Individual and group therapy

Ample discussion occurred in all focus groups about the importance of psychotherapy as a component of treatment. During the focus groups, it became apparent that variations existed within and across FHTs in terms of the types of mental health providers and the types of therapeutic modalities used in therapy. In one focus group, a participant described seeing a psychologist in the FHT and the approaches they used: ‘The focus was cognitive behavioural therapy…. It was a psychologist’ (FG4, P5). In the same focus group, another participant saw a social worker for therapy: ‘[My physician] connected me…with the social worker, and…she's also connected me at one point with a psychiatrist’ (FG4, P7). Focus groups expressed concern that the allotted number of capped therapy sessions was insufficient and may disrupt recovery. ‘There has been sessions that have been helpful, but the consistency hasn't been there…we're actually making progress and then it stops, it actually makes me feel worse…I end up feeling like I lost more than I gained…I’m now lower than I was before we started the whole process’ (FG4, P5). Although the focus groups did not have experience participating in group therapy in FHTs, one participant who completed an individual interview reported benefits of having group therapy in FHTs: ‘I really liked just being able to hear other people's experiences…I’m not alone at least. Like a lot of people there were really supportive’ (I1) (Table 3).

#### 3.3.3 Medication

There was consensus across all focus groups and many individual interviews, about the important role that medication had at one time or another in recovery. ‘The first step she did when I recognized anxiety is a problem…she's connected me with medication’ (FG4, P7). Although most viewed medication as an important component of their treatment for CMDs, there were varying opinions in one focus group whereby some participants felt that their family doctors relied too heavily on medication. ‘I think she would not probe any further because she has given me medication, and I think that's what she knows and she's comfortable with’ (FG4, P9). The dialogue in this focus group continued, ‘It's kind of how they address the blanket issue of mental health…is through medication…that should fix it…. For a lot of people, myself included, it's not really the preferred choice of treatment’ (FG4, P5).

Although there was agreement that psychiatry was an integral part of recovery, not all focus group members had experience with psychiatry. We conducted one focus group in a rural area whereby no primary care teams in that community had psychiatry directly integrated and on‐site, which meant that some patients had to travel long distances to see psychiatry if needed. Those with psychiatry embedded in their primary care team described psychiatry's role as one that mainly did medication consultation or management. ‘I've…been referred to the psychiatrist who has recommended certain drugs that my doctor doesn't feel comfortable administering herself…I went to see the family doctor and she said “I don't feel comfortable giving you these, I'd rather have him prescribe them”’ (FG3, P3). All focus groups expressed surprise by the variations of psychiatry in the primary care teams. Some FHTs had psychiatry on‐site, while others were located off‐site. When off‐site, difficulties of coordination existed between psychiatric and primary care providers. ‘There is no coordination with the family doctor and the psychiatrist. I am the person going to psychiatrist and I’m going to the family doctor, I have to convey the message’ (FG2, P8).

#### 3.3.4 Regular follow‐up

All focus groups viewed regular follow‐up as an essential component of care, yet few participants reported having experienced on‐going follow‐up. One participant described having a primary care physician that initiated routine follow‐up: ‘My doctor follows up with me all the time’ (I4). A focus group learned how meaningful it was for one participant to have their physician proactively follow‐up during a difficult period: ‘She would call me and say, “Hey, how are you doing? Are you ok?”…She's always there for me’ (FG1, P8). Each focus group agreed, however, that it was patients who needed to be proactively initiate follow‐up. ‘You have to be your own advocate, and you need to make sure you follow up, you call people, you can't just leave it in the hands of the doctors because…they're very busy, and…they have a million people like us dealing with them every day’ (FG4, P3). All focus groups agreed that follow‐up needed improvement: ‘There doesn't seem to be a long term kind of thing, where you can feel that somebody's going to check in on me, on my mental health…Instead of waiting until there's a problem…and then it's going to be like “okay let's deal with it”. I think there has to be a long term situation with a check in’ (FG3, P2).

### 3.4 Trusting relationships

There was overwhelming agreement within and across all focus groups and individual interviews that trusting relationships with providers in primary care was essential for mental health care. When asked what high‐quality mental health care looked like, a participant stated:

It looks like people that are providing the service should have empathy, compassion, and understanding, and willing to help people who are suffering from anxiety/depression…And if they don’t have any of these qualities or skills, it doesn’t matter where they complete their education and training from, they won’t be helpful towards the recovery of the patients for the long run. (I2)

Albeit, there was a broad range of relationship experiences that patients had with primary care providers within each focus group.

Having a strong relationship with a physician helped enhance the shared decision‐making process. ‘The relationship that I've built up with my GP…she trusts my judgment… we're usually on the same page in that generally she trusts the conclusion that I come to, because largely a lot of them, we arrived together’ (FG3, P4). Long‐term trusting relationships fostered open communication with physicians and alleviated patients’ fear of judgement:

For me it's the perception of possible shame involved in it. And so like a lot of that is going to end up being the sort of rapport I have with the doctor, if I'm reasonably well convinced that they're going to make me feel like it's a medical condition and not something that I've done wrong (FG3, P4).

Within this focus group, other participants described having very different relationships with their providers in that they needed to ‘earn respect’ of providers in order to be heard, for example, through adopting medical terminology to demonstrate one's knowledge. ‘When you go self‐educate yourself and then you go on and you can like talk the talk, that brings way more respect from the doctors and treats you way more respectfully’ (FG3, P2). Another agreed:

You just get instantly swept under the rug, nobody pays attention to you…if you know the vernacular, they're going to talk to you more respectfully…no one was taking me seriously until you start actually using vernacular that the doctors understand about and then all of a sudden they'll take you super seriously (FG3, P4).

### 3.5 Meeting diverse needs

All focus groups expressed the need for mental health services that meet patients’ diverse needs. The depth of the discussions in each focus group varied, however. For example, one participant in the first focus group explained how the FHT could better address the unique needs of Indigenous patients. ‘I think it would be really great if the [FHT] could have a resident elder for the Indigenous people in this area’ (FG1, P4). Yet, there was minimal exchange between participants following this statement. Meeting diverse needs in mental health care emerged more robustly in the following three focus groups.

Culture emerged in all focus groups and some individual interviews. All focus groups discussed that receiving care from someone from a similar cultural background might be helpful for some patients. For example, one participant found it helpful to see a psychiatrist from a similar cultural background because of his perceived ability to understand her experiences: ‘His understanding of family dynamics, the culture that I come from’ (I2). When asked why it was helpful to have a psychiatrist from a similar cultural background, a participant stated, ‘he speaks my language’ (FG2, P7). The need for providers to understand patients’ cultural backgrounds and how that might influence their mental health‐care experiences emerged in another focus group, ‘I have not disclosed to my family doctor my anxiety…and I think I did not disclose that to her because of my cultural upbringing’ (FG4, P9). The same focus group emphasized that providers needed an understanding of the experiences of immigrants in order to grasp the ramifications on mental health: ‘If you're low income as an immigrant, you don't save enough money compared to the Canadian here…that added to your mental health problem’ (FG4, P1).

There was agreement within focus group two of the importance of gender in mental health care. For example, one participant shared with the group the difficulties she encountered sharing her experiences with a male psychiatrist: ‘I wanted to talk to…a lady psychiatrist…I couldn't talk to him [about] everything I feel’ (FG2, P10). However, gender was not a prominent theme in the other three other focus groups. One topic that was raised in each of the four focus groups was the importance of providers better understanding the impact of socio‐economic status on patients’ mental health: ‘If you're a bit impoverished, then you become anxious. It's a whole cycle’ (FG2, P2).

## 4 DISCUSSION

Understanding the experiences of patients is essential in order to identify how to improve care in a way that is meaningful to patients.^49^ Quality of care is informed by structures—the organization of care—and clinical processes—how care is delivered by providers.^50^, ^51^ Structure is comprised of the physical characteristics of the organization and the staff, whereas processes are the technical care interventions appropriate to the condition and the interpersonal relational interactions that occur between patients and members of the health‐care system.^50^, ^51^ Although all participants in our study spoke about their experiences with the processes of care, it was only through the focus groups that topics related to structure emerged. Participants in our study described their experiences with mental health care across four themes: accessibility, technical care, trusting relationships and meeting diverse needs.

Of utmost importance to our participants was the accessibility of mental health services in primary care for their own recovery.^52^ Integrating mental health providers in the same location as their family physician made a profound difference in the convenience and ease to get care for CMDs when needed. Importantly, our study demonstrates that primary care teams facilitate access to mental health because by reducing patients’ fears of being stigmatized relative to attending organizations known to deliver mental health services exclusively. Stigma limits access to health services.^53^, ^54^, ^55^ Despite expressing how meaningful FHTs are for accessing mental health care, participants raised concerns about the availability^52^ of some services for CMDs.^56^ In particular, participants expressed concerns about capping the number of appointments for psychotherapy and reported that in some cases, the maximum cap was disruptive to their recovery. Additionally, participants noted that there was a lack of available psychiatrists working in collaborative models. This is consistent with reported trends demonstrating the limited availability of psychiatrists, particularly for patients who reside outside of urban areas.^57^

Consistent with what we heard from our participants, primary care physicians are often the first point of contact for patients who experience mental health difficulties.^2^ Overwhelmingly, participants wanted primary care providers to initiate discussions about mental health. Participants in our study went so far as to suggest that screening of CMDs should be implemented in primary care, which is consistent with literature on patient preferences.^58^ Patients find relief having an answer for why something is happening. The Patient Health Questionnaire (PHQ‐9) is one example of a screening tool for depression, although there is on‐going debate about whether or not routine screening for CMDs should be implemented.^59^, ^61^ Undetected and untreated CMDs have measurable and actionable impact on numerous illnesses routinely treated in primary care, such as diabetes for instance.^59^ Integrating mental health services in primary care teams provides a direct pathway from screening to treatment. Our study participants agree with recommendations supporting universal depression screening.^60^ Following our participants’ recommendations, validated tools such as the PHQ‐9 can be used to evaluate and monitor progress, which aligns with measurement‐based care, an increasingly accepted component of quality of care for CMDs.^60^, ^61^ Our findings demonstrate that there is an interest from patients to have systematic approaches to evaluate progress and follow‐up. Greater attention is needed to develop such measurement tools that capture quality and progress in a way that is meaningful for patients.^62^ The current lack of a culture of measurement in mental health care, however, is apparent in the detection and follow‐up of CMDs.^63^

Participants valued having options of choosing the treatment plan that aligned with their personal preference, and primary care teams provided options for patients. Medication was an important component of treatment; however, participants expressed that some physicians were too quick to prescribe medication instead of psychotherapy. The integration of various mental health professionals in the primary care team made individual and group psychotherapy possible^2^, ^18^ and was viewed positively by participants. Focus groups identified regular follow‐up an essential component of care, and described it as particularly meaningful when incorporated in care. Yet, our study demonstrated that receiving regular follow‐up for some patients was difficult. Despite viewing team‐based care positively because it provided patients with treatment options that aligned with their personal needs and preferences, variations existed in the types of treatment modalities and types of providers engaged in their care. The variability in care reported by our participants suggests that there may be an opportunity to improve the consistent adoption of guidelines in the technical care of CMDs in primary care team settings.

Person‐centredness is a core quality of care dimension for PMHs such as FHTs, which focuses on patients’ experiences with whole‐person care, therapeutic relationship and communication.^10^, ^11^, ^12^, ^13^, ^14^ Findings in our study demonstrate that patients highly valued the whole‐person relationship–based care inherent in primary care teams because of the integrated physical, behavioural and mental health realms in care.^2^ Patients emphasized the importance of having a trusting relationship with their provider, communication that aligns with patients’ preferences and being included in shared decision making in a meaningful way. Patients identified trusting relationships as an essential foundation for quality mental health care in our study. For most participants in our study, patient‐centred care was essential for and improved access, which aligns with previous research from providers’ perspectives.^14^ Yet, it was concerning that some patients had to adopt medical jargon in order to gain respect, instead of using language that is most meaningful to them. These types of encounters suggest underlying classism and may further perpetuate inequities in care. Greater attention to the relationship and communication components of mental health care in a person‐centred way will better align care with guiding principles of the PMH model of primary care.^10^, ^11^, ^12^, ^13^

There was an expressed need for better reflection of patients’ gender, culture, ethnicity and language in mental health services offered in primary care teams. For example, some participants expressed the importance of having services in concordant language because it is easier for the patient to relay information and having a shared meaning between provider and patients. Improving services for diverse patients requires that organizations and providers make a commitment to better understand the life stressors and vulnerabilities that contribute to mental health problems, as well as the unique coping strategies and resiliencies that prevent poor mental health and are meaningful for diverse populations.^64^ This level of understanding can help inform culturally safe and culturally tailored mental health care in primary care teams.^65^

### 4.1 Improving quality of care

What emerged in our study is the variability and inconsistency evident from patient feedback collectively. Patients benefit from a structural integration of mental health providers in primary care teams, yet this integration has not yet been fully achieved. There is a variation in the types of and amount of mental health services and types of providers in primary care across Ontario.^22^, ^66^ The extent of these variations was not evident to individual patient prior to focus group engagement, and may not be evident to clinicians without the overall view of the management of their population of patients over time. Undoubtedly, patients in our study benefited from having mental health services integrated in primary care settings yet the variations of experiences described by participants in our study illuminated how there is no consistent foundation of mental health service across primary care settings in Ontario.^2^

Greater attention by policymakers is needed to strengthen integrated collaborative practices in primary care^13^ so that patients have similar access to mental health services across different primary care practices, and smoother continuity of care across sectors. We encourage policymakers to engage with patients to determine how to strengthen mental health services in team‐based primary care. There are various strategies recommended to optimize patient engagement in quality of care including training sessions, clarifying roles, offering stipends and compensation, creating a receptive context and using a buddy system that pairs patients together.^49^ Our study highlights the value of using focus groups to engage patients in an examination of the structural components of care. Through the dynamic exchange of focus groups, patients were able to identify strengths, gaps and inequities that existed for mental health in primary care. Our study raises issues to probe more. Primary care settings treat the majority of individuals facing CMDs; hence, patients must be engaged in order to inform key decisions and drivers of health service issues that prioritize care that is most meaningful for patients.

Co‐researchers on our team brought their lived experiences of living with mental illness, their professional work experiences within and outside of the mental health sector, and the many relationships they cultivated with others who also live with mental illness. Valuing those elements of co‐researchers’ own lived experience has resonance to what research participants voiced in the interviews and focus groups. Future studies aimed at engaging patients for improving quality of care in the design and implementation of initiatives.

### 4.2 Limitations

We conducted this study with patients of one model of team‐based primary care in Ontario, so findings in our study may not apply to all primary care settings. Additionally, our sample did not permit us to gain an in‐depth understanding of the unique experiences of young adults or racialized patients. While focus groups shared some insights about the need for culturally appropriate, relevant and congruent care, we did not probe patients about their perspectives related to racism or sexism in care. Additionally, factors that are not always explicitly mentioned shape people's perception of the quality of care they received, notably experiences of trauma. We also did not probe for examples of care that is more or less trauma‐informed, though care perceived to be of better quality might be more trauma‐informed, and therefore better meeting peoples’ needs.

## 5 CONCLUSION

Integrating mental health services in primary care teams has enhanced quality of care for CMDs, namely by improving accessibility and technical care. Team‐based primary care is an optimal location for mental health services because it is has a foundation of continuity and relationships, which is of utmost importance to patients. Greater attention by policymakers is needed, however, to strengthen integrated primary care so that patients have similar access to mental health services across different primary care practices.

## 6 PATIENT CONTRIBUTION

Service users and individuals with lived experience of depression and anxiety were highly involved in our study. Two individuals with lived experience acted as advisors and participated as co‐researchers in all aspects of the research process including developing interview questions, interpretation of results and preparation of manuscript, and are included as co‐authors on this paper. Additionally, two research assistants responsible for data collection also self‐identified as having lived experience with mental health. All members of our research team have participated in the development of this manuscript.

## CONFLICT OF INTEREST

No conflict of interest for any author.

## AUTHOR CONTRIBUTIONS

RA, MMenear, JS, SD and KM designed the study, prepared funding proposal and secured funding. All authors helped develop data collection tools and sampling strategy. As the research coordinator, ME coordinated and managed data collection, data storage and data analysis. JB and MMuchenje carried out data collection and data analysis. As patient partners, RC and AH were involved in all phases of the study including the following: helping to inform data collection and sampling strategy, interpretation of data and manuscript preparation. RC transcribed audio‐recorded interviews. RA, MMenear, JS, SD, KM, NS, ME, JB, MMuchenje and RC participated in research team‐level data analysis and provided consensus agreement on the findings. AG helped with organization of data and preparation of manuscript. RA, MMenear and AG prepared the initial manuscript draft followed by all authors contributing to the development of sequential versions of the manuscript. All authors edited the manuscript and approved the final version.

## Data Availability

Research data are not shared.
